# Olfaction as a soldier-- a review of the physiology and its present and future use in the military

**DOI:** 10.1186/s40779-017-0119-4

**Published:** 2017-03-15

**Authors:** Palaniappan Ganesh Nagappan, Somasundram Subramaniam, De-Yun Wang

**Affiliations:** 1 School of Clinical Medicine, University of Cambridge, Cambridge, UK; 2 Department of Otolaryngology, Ng Teng Fong General Hospital, Singapore, Singapore; 3 Department of Otolaryngology, National University of Singapore, National University Health System, Singapore, Singapore; 4 Department of Otolaryngology, Yong Loo Lin School of Medicine, National University of Singapore, 1E Kent Ridge Road, Singapore, 119228 Singapore

**Keywords:** Olfaction, Military, Electronic nose, Compensation, Physiology, Injury, Combat

## Abstract

Olfaction is one of our 5 main qualitative sensory abilities. In this review, we have examined the physiology of olfaction from the olfactory receptor to the brain. Through analyzing the physiology of olfaction, we have found that the biochemistry of olfactory nerve stimulation is unique from that of other similar pathways. Upon receiving large amounts of input from the olfactory nerve, the olfactory bulb, followed by several layers of centrifugal and centripetal processing in the brain, has to sort the information from the input as well as integrate it with other inputs from the brain to develop a coherent understanding of the input. We then examined the implications of olfaction in the military, the practical applications of electronic noses and problems associated with injury to olfaction that could affect compensation and combat worthiness of a soldier following injury. In the military, olfaction can allow the army to perform at its best through 4 main methods, namely ensuring olfaction is consistent with other dimensions of perception (ensuring optimal olfaction ability in all soldiers in combat), understanding the impact of different common combat environments on the sense of smell, utilizing odor as a defense mechanism and using olfactory aids when necessary. Electronic noses are olfactory aids that have a large potential in the military ranging from saving lives through the detection of explosives to potential methods for improving combustion efficiency. There are several problems associated with injury to olfaction that should be considered when deciding on compensation and combat worthiness of the soldier following an injury.

## Background

Olfaction, from the Latin word *olfactus*, is the action of smelling or the capacity to smell. Therefore, how does one smell? Olfaction involves the interpretation of chemical odors in the air through a set of human transducer elements that convert the signal into one which can be understood by the various parts of the brain, either for the use of processing in anticipation of a fight or flight response, such as a gas leak in a war zone, or for use in long term memory to remember memorable events and skills, such as the first experience baking a chocolate brownie. This information is transduced through specialized olfactory receptors [1], followed by the olfactory bulb for primary processing and finally the various parts of the brain that process the different aspects and characteristics of the odor. In combat, olfaction is an underutilized sensory ability in warfare that potentially allows for both the detection of an enemy and a tactical advantage in defending one’s location or identifying the enemy [2]. With odorants being propagated through aerosol transmission, the environment plays a major role in an individual’s sense of smell. Odor can be used as a means of protecting one’s self, by using it as a form of stealth, decoy, deterrent and masker. The development of electronic noses as olfactory aids has a large potential in the military, ranging from saving lives through the detection of explosives [3] to potential methods of improving combustion efficiency [4]. With a significant prevalence of mild traumatic brain injuries due to explosions [5] and post-traumatic stress disorder [6, 7] among veterans who served in combat operations, it is necessary to look at the problems associated with injuries to olfaction to assess compensation and combat worthiness of the personnel.

## Overview of olfaction

### First point of contact

The nasal cavity is lined with many receptors. These include receptors for somatosensory sensations (pain, warmth and pressure), with free nerve endings from the ophthalmic and maxillary branches of the trigeminal nerve (CN V), glossopharyngeal nerve (CN IX) and vagus nerve (CN X). However, the qualitative sensations commonly termed odors are mediated solely by the olfactory nerve (CN I). The odor first dissolves into the mucus lining the nasal cavity, after which it then binds to the receptor [8]. Each odor consists of many different types of odor molecules in various combinations. Humans have approximately 450 types of olfactory receptors.

The binding of the odor molecules (similar to ligands) to the olfactory receptor leads to an action potential within the receptor neuron. The secondary messenger pathway here is unique as protein kinase A is not activated, unlike other cAMP pathways in other cells. Olfactory adaptation occurs with sustained and frequent stimulation of the same receptor neurons as elevated Ca^2+^ levels lead to an increased formation of calcium-calmodulin complexes, which inhibit the binding of cAMP to the cyclic nucleotide gated (CNG) channels. It has also been shown that low concentrations of carbon monoxide (CO) increase the activity of the CNG channels [9]. CO activates soluble guanylate cyclase to produce cyclic GMP (cGMP), for which the channel has a much higher affinity compared to that of cAMP.

During a fight or flight response, adrenaline levels and sympathetic stimulation are increased, leading to a heightened sense of smell. Adrenaline increases the stimulation threshold, leading to reduced sensitivity to weak signals, but increases and potentiates strong signals, resulting in an increased awareness of a strong odor [10, 11].

### Primary processing of the signal

Primary processing of olfactory signals occurs in the olfactory bulb [12]. The unmyelinated axons from the olfactory receptor cells (ORCs) ascend through the perforations of the cribriform plate of the ethmoid bone to synapse at the olfactory bulb [13]. The unmyelinated axons converge on the outer layer of the olfactory bulb within small structures (diameter <50 μm) called glomeruli (glomerular layer) [14]. From here, they form synapses with second-order neurons (mitral and tufted cells) located on the inner layer of the olfactory bulb. The mitral cells project these signals to higher brain centers within the primary olfactory cortex (including the anterior olfactory nucleus, olfactory tubercle, piriform cortex, the lateral entorhinal cortex and the periamygdaloid cortex), allowing for multiple signals to be processed to form a synthesized olfactory perception. A large degree of convergence occurs where approximately 25,000 axons synapse onto approximately 25 mitral cells, with each mitral cell receiving signals from multiple glomeruli. Mitral cells also project to periglomerular cells and granular cells that inhibit the mitral cells surrounding it, providing lateral inhibition. This facilitates better discrimination between signals, improves specificity and produces a better signal-to-noise ratio, which clinically translates to better smell perception.

Interbulbar communication (occurring *via* the anterior commissure) and complex intrabulbar communication are present between the interglomerular cells, periglomerular cells and granule cells, which help create a spatial map [15]. There are also collateral projections from the mitral and tufted cells. The olfactory bulb receives centrifugal fibers from higher centers of the brain, including those to which it projects. Most of these fibers terminate in the external plexiform layer and the granule cell layer of the bulb, as these are the areas that the mitral cells can be best influenced, as observed through the positions where lateral inhibition occurs. However, terminations do occur in all layers except the glomerular layer [16].

### Final stage of processing

**Fig. 1:**
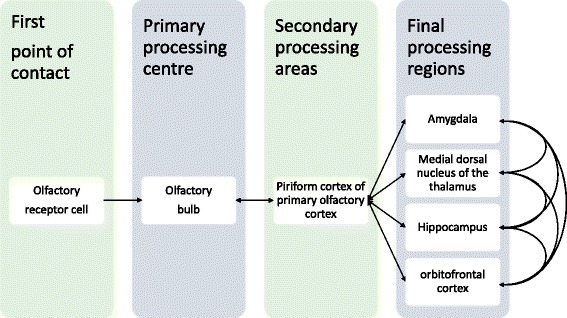
A simplified pathway of the major regions through which an olfactory signal pass. Arrowed lines indicated a one-way direction, while bidirectional lines indicate bidirectional signal transmission. The olfactory signal is processed through these major regions, integrating it with other signals

Beyond the olfactory bulb, the neural pathways through which an olfactory signal passes through the brain are numerous and varied. The main areas for the processing of these signals are the amygdala, hippocampus and orbitofrontal cortex. The routes taken to these main areas run primarily through the piriform cortex of the primary olfactory cortex and the thalamus [17]. These serve as processing areas for all sensory information. According to Shipley and Reyes [7], the entorhinal cortex provides the most direct access to the hippocampus from the olfactory bulb, which is important to note, as the shortest route does not make it the default route for signal propagation. The entorhinal cortex also receives innervation from the amygdala. A simplified version of the pathway is shown in Fig. 1.

The mitral cells leave the olfactory bulb in the lateral olfactory tract, which then forms synapses with various regions of the primary olfactory cortex: anterior olfactory nucleus, olfactory tubercle, anterior and posterior piriform cortex (APC and PPC), lateral entorhinal cortex and the periamygdaloid cortex. The APC is known for determining the chemical composition of the odor molecules. The PPC is known for categorizing odors and making comparisons between odors in a concentration-independent manner [18]. The anterior olfactory nucleus, through the anterior commissure, inhibits the contralateral olfactory bulb.

The piriform cortex then projects to the amygdala, medial dorsal nucleus of the thalamus, hippocampus and the orbitofrontal cortex. There are many direct and indirect connections between these regions of the brain.

The amygdala is involved in associated learning, where the odors help to serve as rewards or punishments in the associative learning process. This means that odors that are associated with positive states reinforce behavior that leads to more of the positive state and vice versa for the negative states. Brain imaging studies have found that activation of the amygdala correlates with pleasant and unpleasant odors, reflecting the link between odors and emotions [19].

The hippocampus, similar to the amygdala, assists with the learning process as well. In addition, the hippocampus is also associated with episodic memory. This is where an odor leads to the retrieval of a memory from a specific point in time or place [20].

The orbitofrontal cortex integrates olfactory signals and taste with each other, as both signals lead to the same region. This results in the coupling of smell and taste, making food and beverages we consume much more appetizing and less so when our smell is impaired with a common cold, for example. Odor perception and discrimination also occurs here as part of a spatial odor map to recognize certain specific odors. The orbitofrontal cortex also projects to the anterior cingulate cortex, allowing it to play a further role in appetite [21].

Temporal coding and spatial maps allow humans to distinguish between the many odors. Temporal coding occurs when neural signals are sent with specific spike patterns and spike rates. The spatial excitation map for different odors varies largely within the olfactory bulb itself. This is due to the versatility of the neurons that carry the different types of odor signals, as well as a complex network of intrabulbar and interbulbar connections. This forms complex logic circuits, allowing for a greater processing capacity, in order to identify the odors in question. As each nostril has its own input into the brain, the bilateral activity results in a greater depth of signals due to perceptual rivalry [22].

## Implications of olfaction in the military

Olfaction is one of the 5 main sensory signals that allow us to comprehend and interact with our environment. There are four main aspects to consider when applying a sensory signal in the field: whether the odor signals are congruent with other dimensions of perception, the distance between the odor source and the target, the type of environment, and one’s own safety [23]. For a soldier to perform at his best in a combat situation, he would need to utilize all of his sensory signals to their maximum ability. Therefore, to analyze how olfaction can be affected is essential to the development of solutions or precautions to prevent and limit harm caused to the soldier.

### Congruency with other dimensions of perception

A person’s olfactory ability declines over time [24]. Furthermore, after suffering from mild traumatic brain injury, the likelihood of loss of olfaction tends to increase as well [25]. To be able to understand whether a soldier is still fit for battle requires a test of olfaction along with other fitness and medical tests before a mission. This can be done through olfactory tests [26] such as the University of Pennsylvania Smell Identification Test (UPSIT) and Sniffin’ Sticks. These tests will serve as good screening tools to identify soldiers with weakened olfaction.

### Common types of environments and their impact on olfaction

**Fig. 2:**
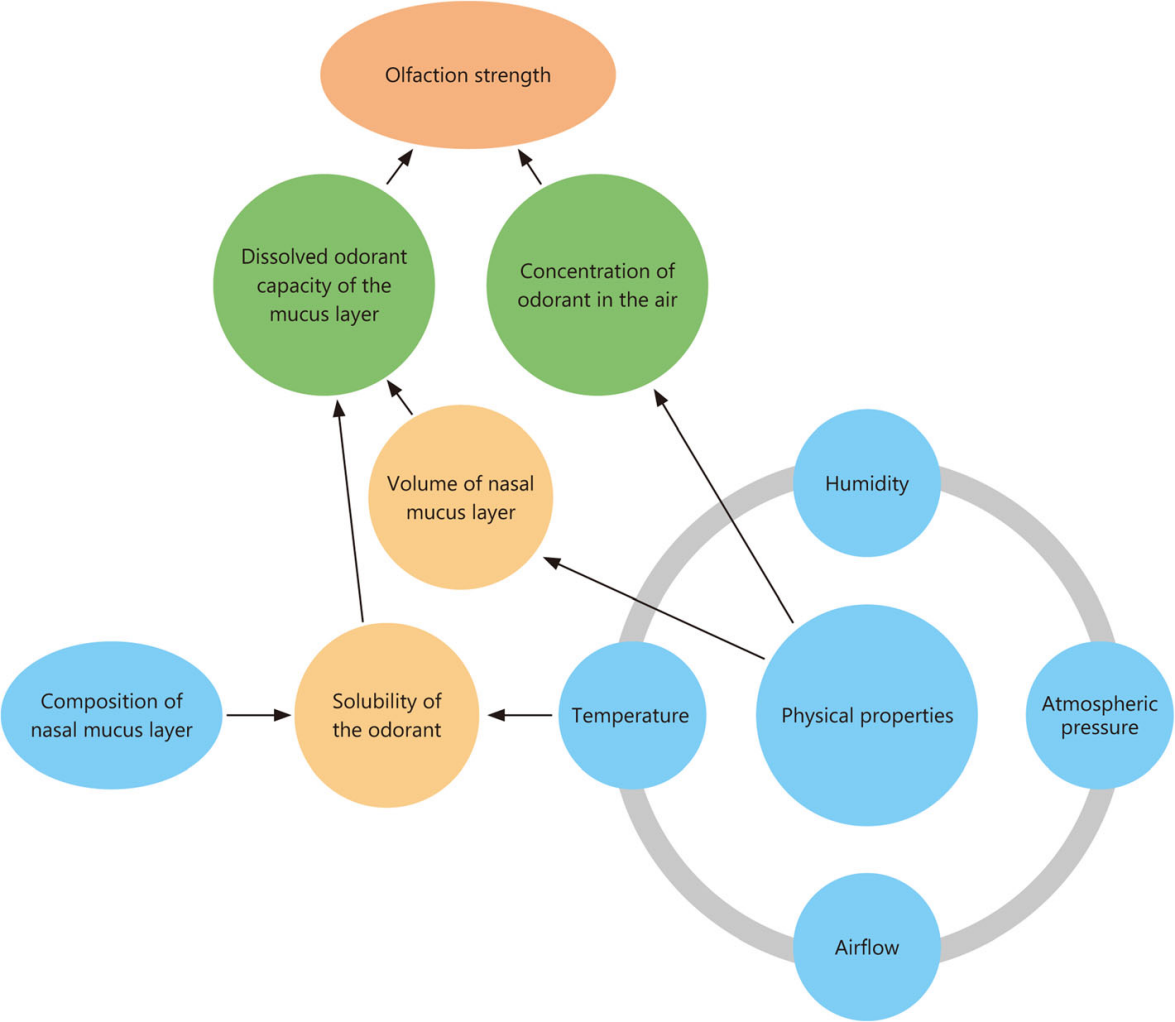
Factors that affect the olfactory signal. The olfactory signal strength one receives is dependent on several factors as illustrated in the figure. Temperature, humidity, atmospheric pressure and airflow all relate to one another and collectively exert an effect on the evaporation rate of the nasal mucus layer as well as the odorants themselves. This forms the basis on which the other variables act to provide the observed olfactory signal strength

Olfaction is dependent on the concentration of vapor in the air and the capacity for it to dissolve into the nasal mucous lining. Maximum olfaction is achieved through a combination of high concentration of vapor in the air together with high solubility of the odorant molecule into the mucous lining. With details from Table 1, we can also construct Fig. 2 to give a representation of how the different types of environments can affect olfaction by looking at the factors affecting the concentration of odorants in the air and the factors affecting the dissolved odorant capacity in the nasal mucosal lining.

**Table 1:** Some examples of biomes and their environmental features which would influence olfaction

Biomes	Humidity	Atmospheric pressure	Temperature	Airflow (Wind Speed)
Siberian Tundra	Extremely Low	High	Extremely Low	High
Sahara Desert	Extremely Low	High	High	High
Mount Everest	Low	Low	Low	High
Argentinian Grassland	Moderate	High	Moderate	Variable
Alaskan Forest (Taiga)	High*	High	Low	Variable
Tropical Borneo Rainforests	High	High	High	Low

The data [116–118] used are a general interpretation of the climate of these regions, which averages out the weather patterns covering a group of areas over a significant period of time. The values High, Moderate, Low and Extremely Low are all relative to each other. The environments here were selected to provide a basis to illustrate examples of places with differing humidity, atmospheric pressure, temperature and airflow. Relative Humidity here is highly variable, as changes in temperature can affect the carrying capacity of the air, affecting its relative humidity. * Low evaporation rates and low temperatures lead to high relative humidity

There are 4 main factors affecting the concentration of odorants in the air: temperature, atmospheric pressure, humidity and airflow. With higher temperatures [27], the vapor pressure is increased, thereby increasing the rate of diffusion of the odorants, thus increasing the overall detection range. The opposite is true for lower temperatures. At lower atmospheric pressures, the vapor pressure of odorants increases, allowing the concentration of the odorants to increase [28]. However, this is countered by higher evaporation rates of the nasal mucosal lining, as explained below. An increase in humidity has been shown to increase olfactory detection of odorants, although the mechanism is unclear. It has been speculated that increased humidity increases the capacity of the air to carry odorants [28]. With a higher airflow, there would be a greater volume of odorants in the air, as well as an acceleration of the odorant’s travelling speed by the wind. However, the concentration is dependent on the direction and path of the wind. It would be advantageous if the wind was blowing towards the individual but a disadvantage if the wind was blowing away from the individual.

There are 2 main factors affecting the dissolved odorant capacity within the nasal mucosal layer: solubility of the odorant and the volume of the nasal mucosa.

The solubility of the odorant is affected by the composition of the nasal mucous layer and the temperature of the nasal mucous layer. Further research could be done to improve the solubility of odorants into the nasal mucosal lining. Methods by which this could be possible include pharmaceutical means similar to nasal drug administration [12]. A higher temperature would also increase the solubility of most odorants [27].

The volume of the nasal mucous layer is affected by temperature, atmospheric pressure, humidity and airflow. A higher temperature increases the evaporation rate of the nasal mucus layer, reducing the volume of the nasal mucus layer present [27]. A lower atmospheric pressure leads to a higher vapor pressure of the nasal mucus layer and thus a higher evaporation rate, reducing the nasal mucus layer, overall leading to the impairment of olfactory sensitivity at a lower atmospheric pressure [28]. Higher humidity lowers the vapor pressure, thus retaining or even increasing the nasal mucus layer, depending on the rate of mucous production [28]. Higher airflow causes a reduction in the nasal mucus layer through increased evaporation. A possible explanation to why the volume of the nasal mucosa is important in olfaction is that it is always changing (due to secretion and evaporation), thereby affecting the concentration and maximum capacity of odorants that can be dissolved. For maximal binding of ligands to receptors, there needs to be a high concentration and adequate amounts of odor molecules in the nasal mucosa to be able to interact with the olfactory receptors, thus requiring a relatively constant range of nasal mucosa volume. If the nasal mucous layer is too thick, the odorants may take longer to diffuse to the receptor, and in the process, the concentration of the odorant would be greatly reduced. This increases the delay in detecting odorants and increases the odorant threshold. If the nasal mucosa is too thin, not enough odor molecules are able to dissolve in the mucosa, limiting the maximal binding of the odorant to the receptor (as the ligand, odorant, is the limiting factor). Further research is necessary to understand the relevance of the volume of nasal mucosa in olfaction.

Some publications have examined the effect of temperature at the receptor level, considering the effects of temperature on the biology of *Drosophila melanogaster.* A higher temperature was found to lead to a higher olfactory response recorded [15]. Other possible impacts of temperature on the biology can occur at the cellular level and the molecular level. At the cellular level, there could be effects on the nervous conduction and velocity, leading to possible conduction delays [29]. At the molecular level, heat-shock proteins [30] and cold-shock proteins [31] could lead to biochemical reactions that could have an effect on olfaction as a whole.

Therefore, in order to gain the best possible advantage in the field using just the human nose, one would have to keep their nose relatively warm and moist to better detect any odorants in the environment.

### Odor as a defense

To move stealthily, odor should also be shielded from the enemy to protect one’s safety. There are 4 main methods by which this can be carried out [2]: 1) stealth operations, 2) decoy, 3) deterrent and 4) masker.

The goals of stealth operations are “to develop novel means to minimize detection of intended activities through sensory diversion and by presenting false information to the enemy about the surrounding environment” [32]. Such operations would include methods for hiding one’s presence to divert enemy attention from one’s actions in order to mislead the enemy regarding one’s strength or intentions. Four factors need to be considered while using odors in psychological operations [33] — consistency, distance (proximity of target), environment (metrological factors — wind) and OPSEC (operations security — activity odors should be masked or eliminated). However, more research into the olfactory processes at the cognitive level is necessary to fully utilize odor in stealth operations.

Decoys involve distracting the enemy with the use of another more obvious signal to direct the attention of the enemy away from the actual operations. In the past, decoy operations using odors have been successfully carried out. An example would be the Allied Forces invasion of Normandy, where simulated odor was used in deceiving Operation Titanic [34]. The use of decoys can stymie even the best surveillance systems, providing a good military advantage against the enemy and posing a potential problem for defending against enemies [35].

Deterrents are important in order to keep enemies away from one’s operations. Odors that are involved in this purpose would be ones that dominate the environment. Such methods are being used in the civilian world, such as using fox urine or its components [36] to spray Christmas trees [37]. In the cold, its smell is imperceptible. However, once brought into a warm room, its odorants diffuse at a much faster rate, acting as a strong deterrent to use such trees. As a pest control measure, a combination of deterrents and decoys have been employed as a ‘stimulo-deterrent diversion’ as a more effective method to protect crops from pests [38]. This could be applied in the military where a number of these methods could be used together to potentiate the benefits of odor to protect one’s interests.

Maskers are neutral odors that are strong and natural enough to mask an existing smell to make that odor blend into the background and become undetectable [39]. Another possible mechanism that may have military applications is the use of two odors that compete for recognition, which activate and deactivate the same neuron simultaneously, thereby inhibiting the signal production and leading to a lower combined perceived intensity [40] (also known as counteraction). Some hunters use activated carbon-containing personal items to mask [41] the hunter’s scent, allowing for improved hunting.

Therefore, a combination of these four methods can be used to provide an effective odor shield to protect oneself from the enemy.

## Use of olfactory aids on the battlefield

### Dogs

Dogs have been used as aids in the military since World War 2 to detect mines [42]. Dogs have adapted to become one of the best living examples of an ideal smell detector. They have developed an olfactory epithelium 20 times larger than humans [43] with 100 times more receptors per square centimeter [44]. Dogs also have a larger olfactory bulb allowing them to have more capacity to process signals from their wider repertoire of olfactory receptors. With more olfactory receptors present in the olfactory epithelium, dogs are undoubtedly able to detect odorants at much lower concentration than humans [43]. In addition, dogs only have sweat glands on their paws. Their hair coats limit heat loss through vasodilation; thus, vasodilation is restricted to the mouth, nose, back of ears, paws and other areas with less hair [45]. This allows for the nose to be consistently moist and warm, allowing for maximum solubility of odorants. Therefore, dogs serve an important role in detecting bombs, concealed mines and drugs, and other illegal substances.

### Electronic noses

Electronic noses have now been developed to provide an alternative to using living beings to detect smells [46]. An electronic nose has a rather high sensitivity and specificity, similar to that of humans, when tested with various odorants. Its small size is also a significant advantage. However, its capabilities are currently limited by the number of odorants it can detect, but this will likely expand with time. Electronic noses also have the added benefit of not placing any lives in danger while still delivering equivalent or superior results. Electronic noses have many applications that can be used in the military to enhance battle readiness of soldiers in the field. The applications of electronic noses relevant to the military can be grouped into 5 main areas: Detection of explosives, environmental monitoring, medical diagnostics and health monitoring, automotive and aerospace applications and food and beverage quality assurance. An organized summary is presented in Table 2.

**Table 2:** Electronic nose applications

Electronic nose applications	Techniques currently being employed	Benefits in the military	Disadvantages
Detection of Explosives (Landmines)	• By humans with simple metal detectors • Human-dog teams (faster)	• Save human lives •Work around the clock • Improve security for humans (good warning tool) • Conserve marine ecology	• Need to outperform dogs • Require high sensitivity and high selectivity • Need to be low maintenance • Need to be robust
Environmental Monitoring	• Traditional Methods: Olfactometry measurements • Interpreted by qualified humans and identification with analytical instruments	• Detect toxic chemicals • Detect smoke [50] • Indoor air quality [51–54] • Automotive ventilation control [55, 56] • Measure water quality	• Sampling is difficult • Needs to be representative of the surroundings • Need to be adjustable to standardized humidity and temperature.
Medical Diagnostics and Health Monitoring	• Olfaction to help in diagnosis largely ignored [62] with availability of modern diagnostic techniques	Tested with • bacteria [69, 70] • metabolic diseases [9, 74, 75] • monitoring hemodialysis [76].	• Need to reduce the false-positive and false-negative rates • Need to understand the impact of common factors (nutrition [77, 78] and medication [79]) on humans
Automotive and Aerospace Applications	• Currently conceptual • Used in NASA’s STS-95 flight	Beneficial uses: • Monitor exhaust to feedback to engine for higher combustion efficiency • Monitor the cabin air for passenger safety	• Need for devices that are cheap and small • Need to be able to detect a variety of relevant odors for particular applications.
Food and Beverage Quality Assurance	• Adherence to use-by dates • Use by dates determined by experimental research	• Able to ensure whether food is edible in times of crises and different environments • More sensitive and accurate [81]	• Spoilage compounds differ with different types of food and beverage [85].

Detection of explosives (primarily landmines) is one of the major applications of electronic noses in the military. The current applications include demining with simple metal detectors or human-dog teams. The former proceeds at 200 m/day, while the latter allows 2–4 km/day to be cleared. It is unclear whether dogs detect the pure explosive or impurities associated with the explosive [47]. ICx Nomadics have created the first known real-time sensor capable of detecting chemical signatures from underwater unexploded ordnance (UUXO) [3]. The electronic nose technology would save human lives, be able to work around the clock to demine without fatigue, and improve security for all humans. The system developed by ICx Nomadics has been identified as one of the best currently available detection devices using chemical sensors based on the amplification of fluorescent-conjugated polymers [48] with a vapor detection limit of 1 fg/ml, as opposed to 1 ng/ml with HPLC-UV [49]. This would prove to be a very good warning tool for soldiers that may be heading into a zone with explosives, alerting them to it and allowing for the localization and disposal of the explosive. The removal of UUXO not only improves the security of the area but also eliminates a significant source of toxicity to local marine organisms. To summarize the characteristics of the ideal device, these would include its ability to outperform dogs, exhibit high sensitivity to the explosive vapors, demonstrate the ability to selectively detect only relevant vapors among the clutter while maintaining low false-positive activation, have low maintenance costs, and be structurally robust while in use in the field.

Environmental monitoring is another application for the electronic nose in the military. Emission ratings and detection currently use traditional methods, including olfactometry measurements realized by a human panel, with qualification and identification using analytical instruments. These are not appropriate for on-site, real-time and continuous operation due to the high operating costs. In the military, an electronic nose can be used to detect toxic chemicals and smoke compounds [50], determine indoor air quality [51–54], control automotive ventilation [55, 56], and measure water quality of an emergency water source (specific examples of detection of water quality: residues of cyanobacteria [57] and pesticides [58]; general examples: water pollution [59] and wastewater samples [60, 61]). The possible benefits of an electronic nose in terms of monitoring the environment are numerous, as it can be used in camp, in the field and in survival situations. However, at present, its practical applications are limited as the samples need to first be representative of the surroundings and then be measured at the same humidity and temperature for it to have a standardized and reliable result.

Medical diagnostics and health monitoring using an electronic nose can provide new and possibly better ways to detect diseases. The currently available modern diagnostic techniques provide more precise information utilizing the physical, chemical and microbiological methods of observation, superseding the role of the subjective odor perception of the physician. However, this would ignore many pieces of information on the overall health condition of the patient, for which the physician is undoubtedly better trained to ascertain [62]. The electronic nose technology has already been tested for a variety of diseases and samples, including identifying bacteria (in leg ulcers [63], vaginal swabs [64–66], upper respiratory tract [67, 68], mycobacterium tuberculosis [69, 70] and urinary tract infection [71–73]), the diagnosing of metabolic diseases (in diabetes [9, 74] and renal dysfunction [75]) and monitoring hemodialysis [76]. The ability for the electronic nose to detect such a wide range of diseases at present is encouraging. As research progresses, it is foreseeable that the electronic nose could become a simple and efficient method to detect a wide range of diseases effectively. For the electronic nose to be applicable in the medical environment, its diagnostic accuracy would need to be improved (by minimizing false-positive and false-negative rates). We also have to factor in the heterogeneity and diversity of humans, with the interplay of complex factors such as nutrition [77, 78] and medication [79] on the sample set with respect to the results obtained with the electronic nose.

The use of an electronic nose in the automotive and aerospace divisions of the military shows promise. Currently, its use is primarily conceptual with the main application in NASA’s space shuttle Flight STS-95. The possible automotive applications of an electronic nose would be to monitor exhaust fumes, providing feedback to the engine to improve combustion efficiency. It could also be used to monitor the cabin air for passenger safety, determining early when the inside air may be hazardous due to possible leakage of oil or coolant into the air intake. Aerospace applications would be relatively similar to the automotive industry, except that passengers would be in enclosed cabins where the composition of the air needs to be carefully monitored, especially in the event that the plane is carrying volatile and hazardous substances [4]. More research needs to be done into electronic noses to develop devices that are cheap and small, yet able to detect a variety of relevant odors for its various applications.

Food and beverage quality assurance in the military is essential especially in times of food crises, where food supply is scarce and would need to be kept for prolonged periods of time. Currently, the shelf-life of food is based on adherence to use-by dates, which are centered on previous experimental research into the length of time that the food and beverage can last. The benefits of the electronic nose lies in the detection of the quality of food, especially in the event of a food crisis, in different environments (temperature and humidity [80]). Examples of where electronic noses have been proven to be better detectors than humans include the BloodHound BH-114 (which detects spoilage as well as fungal species in a bread analogue) [81] and CO sensors for haddock fillets [82]. What is challenging here is that spoilage compounds may differ depending on the type of food. In fish for example, spoilage compounds [83, 84] differ between species, parts of the fish [85] and treatment of the fish upon capture [86, 87]. Sensors may not be sensitive enough to provide the relevant information, such as the NH3 sensor in detecting trimethylamine (TMA) concentrations (CO sensors are better here [88]).

The future of electronic noses relies on the number of different types of odors an electronic nose can pick up, which can be improved by increasing the capabilities of the sensors [89] and improving the algorithm for signal processing to identify the type of odor (using intelligent and statistical pattern analysis) [90].

## Problems associated with injury to olfaction in the military

Military personnel being deployed to combat areas could be exposed to toxins and chemicals, which when exposed to the olfactory epithelium, can lead to olfactory disorders [91]. Examples include Halabja chemical attack (1980, including mustard and nerve agents [92]) and the Ghouta chemical attack (2013, Sarin). Exposure to such chemical toxins can lead to many deteriorating conditions, and the reactions can be categorized under three main classes of toxins: 1) nerve agents, 2) blister agents and 3) phosgene [11, 93]. Under low vapor pressure of such agents, rhinorrhea may occur [93]. However, these agents can also burn at low concentrations. Burning of the columnar olfactory epithelium of the nose can lead to hyposmia or anosmia. Smell dysfunction can also be caused by several other factors (such as head trauma, upper respiratory tract infection, rhinosinusitis and chronic rhinitis) [94].

Due to the anatomy of the olfactory nerves, soldiers with head trauma [95] could potentially suffer from olfactory dysfunction [96] in the event of disruption of the cribriform plate or surrounding areas. Soldiers that complain of head trauma-related olfactory dysfunction typically have anosmia and rarely regain normal olfactory ability. The appropriate MRI protocol can be used to observe the damage to olfactory-related brain structures in such patients [97]. Moreover, olfaction appears to be the most sensitive physical examination biomarker for residual neurological dysfunction due to mild traumatic brain injury [1].

Smell can be associated with the memories [20] and pain sensations felt at a particular point in time, leading to episodic memory and an association of the smell and pain felt. In Post-Traumatic Stress Disorder (PTSD), certain smells have the potential to precipitate traumatic memories with strong emotional components [6]. The smell of these toxic gases can be both unique and ubiquitous. Soman, a nerve gas, smells like camphor [98]. Phosgene oxime, a blister agent, smells like freshly mown hay [99]. Therefore, exposure to similar smells in daily life might trigger memories [100] from the past and their associated emotional components, leading to PTSD. There is currently a possibility for treatment of PTSD with the help of olfaction and virtual reality therapies [101].

Depression can result from olfactory damage. Research has shown that removal of the olfactory bulbs in rats leads to dendritic reorganization, disrupted cell growth and decreased neuroplasticity of the hippocampus, along with behavioral changes similar to those observed in people with depression [102]. This shows the reliance of the hippocampus on stimulation from the olfactory bulb to retain its neuroplasticity and active cell growth.

Smell dysfunction has the potential to adversely affect the quality of life of military personnel. Olfaction has been suggested to converge with other special sensory inputs such as the sense of taste and vision in the orbitofrontal cortex [103] to enjoy the food one is eating, for example. Loss of this convergence and potentiation leads to a less gratifying meal [104]. Olfaction is also involved in creating episodic memories and processing remote associative olfactory memories [105]. Thus, with olfactory dysfunction, memories have a reduced association to olfaction and are less detailed in that aspect.

Furthermore, in combat situations, smell is a special sensory ability that acts as a warning signal [106] to alert personnel to any dangers [107] nearby, especially if the danger is inaudible, invisible or not in the direct line of sight. Any olfactory dysfunction could pose as a handicap to the soldier in such a situation, leading to potential harm to himself.

All of the above factors need to be assessed upon evaluation of the injury in order to assess the level of compensation and the combat worthiness of the soldier. Studies on the impact of olfactory impairment on quality of life and disability have shown that patients reporting persistent olfactory impairment after previously documented loss showed a higher level of disability and lower quality of life than those with perceived resolution of an olfactory compromise [108]. In particular, their ability to detect smoke, natural gases or other toxins in the environment was affected [109], and they had a higher propensity to develop depression [110]. A study on olfactory impairment in an adult population that focused more on the general adult population with emphasis on dietary choices and quality of life found that olfactory impairment had no effect on quality of life [111]. However, due to the nature of the participants of this study representing the general adult population, the prevalence of olfactory impairment was low and may not have included a representative sample. Based on the current evidence, it is probably preferable for soldiers in combat not to have anosmia, while it is preferable for those in special forces to have full olfactory ability or not more than minimal hyposmia. Further studies still need to be done specifically on soldiers who have been in combat to produce a more representative sample in order to arrive at a more definitive conclusion. We also suggest that a test for olfaction (using the methods mentioned above) upon admission into the military should be conducted, which could be useful in providing initial data against which subsequent data could be compared in the event of potential compensation for an injury later in their career, as well as a potential diagnostic marker for anosmia and other neurodegenerative disorders.

## Neurodegenerative diseases and its impact on olfaction

Olfactory dysfunction is often present as a symptom of neurodegenerative disease. It is found in as high as 100% of Alzheimer’s disease cases, 96% of the frontal variant cases of frontotemporal dementia, 90% of Parkinson’s disease cases and 15% of vascular dementia cases [112]. According to Duff [17], discriminating between patients with Alzheimer’s disease from those with vascular dementia and major depression can be easily achieved through olfactory testing with a high specificity and sensitivity, as vascular dementia is not usually associated with olfactory dysfunction. Furthermore, in Parkinson’s patients, olfactory dysfunction occurs before motor weakness is observed, making it a good early screening tool. On the other hand, the current limitation of utilizing olfactory dysfunction to diagnose neurodegenerative diseases is the inability to conclusively differentiate between them (e.g., cases of depressive pseudo dementia, Lewy body disease, or dementia of mixed etiology) [107, 113]. While neurodegenerative diseases involve the olfactory tracts early in the disease process, the reverse effect has also been shown to occur with depression occurring in olfactory bulbectomized rats [102]. Young soldiers rarely tend to present with neurodegenerative diseases; however, the early diagnosis of neurodegenerative diseases should not be ruled out due to the many unforeseen circumstances that may occur during wartime.

## Current research into olfaction in the military

There are several publications related to olfaction in the military that focus on the use of olfaction as a tool for detection, treatment or both. Olfaction has been found to be useful in the detection of UUXO [3] and as a means to avoid detection in ‘Olfaction Warfare’ [2]. As a form of treatment, olfaction has also been found to be the most sensitive physical examination biomarker for residual neurological dysfunction due to mild traumatic brain injury [1]. Due to the close association between olfaction and memory, there is a possibility of using olfaction along with virtual reality as a form of PTSD therapy, which would be very useful in the military given the prevalence of PTSD [6, 101]. There was also a publication in 1973 on the potential applications of olfactory research in man with relevance to the military [14]. As a form of training, the immersion of participants into virtual environments has not shown to be enhanced with the use of olfaction [114]. However, another publication demonstrated that odors can become readily associated with emotions and can thereby influence behavior [115], indicating a possible avenue for training a soldier’s response in combat. There is unfortunately not as much research into olfaction in the military setting as we would have liked there to be. This could possibly be due to limited resources, ignorance or restrictions with respect to military research.

## Conclusion

Olfaction is akin to the ability to detect the chemical nature of the surrounding air, transducing the signal into one that the brain can understand — the perception of smell. It can be used as a form of defense, detection, diagnostic method, and possible treatment options in addition to many other applications. Olfaction plays a major unseen role as an innate alarm. It could also be used as a means to gain a significant tactical advantage over the enemy in a battle field, as it is still a relatively underrated and underdeveloped but potentially powerful qualitative sensory ability. Pursuing further research into the exact cut-off point of olfaction ability in the military would be fruitful, especially in the special forces, in order to ensure that the lives of soldiers would not be put in jeopardy. To date, the literature has shown certain aspects of potential uses of olfaction in different fields, but this study offers a review of the current publications with regards to the wide range of current and potential uses of olfaction in the military, as well as the significance of the olfactory sense along with its physiology. This is important for the further utilization of olfaction in the military for research and practical purposes.

## Abbreviations

APC: Anterior piriform cortex
cGMP: Cyclic guanosine monophosphate
CNG: Cyclic nucleotide gated
CO: Carbon monoxide
OPSEC: Operations security
ORC: Olfactory receptor cells
PPC: Posterior piriform cortex
PTSD: Post-traumatic stress disorder
TMA: Trimethylamine
UPSIT: University of Pennsylvania smell identification test
UUXO: Underwater unexploded ordnance
